# Precipitation as the Primary Environmental Driver of Saproxylic Fly Diversity (Diptera: Bibionomorpha and Tipulomorpha) in Forest Ecosystems

**DOI:** 10.3390/insects16111109

**Published:** 2025-10-30

**Authors:** Ina Gorban, Giedrius Trakimas, Laurynas Stasiukynas, Aistė Lekoveckaitė, Arūnas Samas, Virginija Podėnienė

**Affiliations:** 1 Institute of Biosciences, Life Sciences Center, Vilnius University, LT-10257 Vilnius, Lithuania; giedrius.trakimas@gf.vu.lt (G.T.); laurynas.stasiukynas@gmc.vu.lt (L.S.); aiste.lekoveckaite@gmc.vu.lt (A.L.); arunas.samas@gf.vu.lt (A.S.); virginija.podeniene@gf.vu.lt (V.P.); 2 Institute of Life Sciences and Technology, Daugavpils University, LV-5401 Daugavpils, Latvia

**Keywords:** diptera, dead wood, climatic variables, saproxylic

## Abstract

**Simple Summary:**

Saproxylic nematoceran flies (Bibionomorpha, Tipulomorpha) are an important group of forest ecosystems, contributing to decomposition and nutrient cycling. We investigated how climatic factors—precipitation, temperature, and relative humidity—affect their communities in three Lithuanian forest reserves over five years. Using trunk-emergence traps, we collected 793 specimens from 10 Diptera families. Statistical analyses showed that precipitation was the only significant factor positively associated with species richness, abundance, and diversity, while temperature and relative humidity had no significant effects. These findings emphasise the importance of precipitation and suggest that saproxylic fly communities may be vulnerable to increasing drought frequency under climate change.

**Abstract:**

Saproxylic insects of the infraorders Bibionomorpha and Tipulomorpha (Diptera) are ecologically important components of forest ecosystems, associated with dead wood and contributing to nutrient cycling and food webs. Despite their diversity, the influence of climatic variables on saproxylic nematoceran communities remains poorly understood. This study examined the effects of precipitation, air temperature, and relative humidity on the abundance, species richness, and diversity of saproxylic nematoceran flies in three Lithuanian nature reserves from 2016 to 2020. In total, 793 specimens representing 10 families were collected using trunk-emergence traps. Statistical models showed that precipitation was significantly positively associated with species richness, total abundance, and Shannon’s diversity, whereas temperature and humidity were excluded as non-significant predictors. These results highlight precipitation as a key environmental driver shaping saproxylic fly assemblages and suggest potential sensitivity of these communities to reduced moisture availability under future climate change scenarios.

## 1. Introduction

The order Diptera, commonly known as true flies, represents one of the most species-rich insect groups, with over 40,300 species documented in the Palearctic region [1]. Within this order, the infraorders Bibionomorpha and Tipulomorpha include a wide variety of species in forest ecosystems, many of which are associated with decomposing wood. Dead wood, including fallen trees, branches, and stumps, is a crucial habitat that provides resources essential for larval development [2]. It supports a wide array of trophic groups among Diptera, including xylosaprophagous, mycetophagous, and zoophagous taxa [3].

Xylosaprophagous larvae feed directly on decaying wood, while mycetophagous species rely on fungi colonising decomposing woody material. Zoophagous species, in turn, prey upon other organisms inhabiting the same microhabitats. Saproxylic nematoceran flies occupy a variety of microhabitats within dead wood, such as the space beneath bark, sap flows, and the moist layers beneath moss [4]. Their diversity can vary not only among tree species but also across different stages of wood decay. Species belonging to Sciaridae and Mycetophilidae, for instance, often show the highest abundance and diversity during intermediate decay stages, when the bark remains intact and fungal mycelium has already penetrated the wood [5,6]. Moreover, even species not directly involved in wood decomposition use deadwood as a site for pupation and overwintering, highlighting its broader ecological importance [7]. Thus, microhabitat heterogeneity within dead wood is a key factor maintaining the diversity of saproxylic Diptera [8,9].

Among the abiotic factors influencing saproxylic fly assemblages, precipitation and overall moisture availability play a particularly critical role, affecting both larval development and adult distribution [8]. Nematoceran larvae inhabit the moist layers beneath bark or within decaying wood, thriving in environments with stable water content [10]. Some families, such as Mycetophilidae, adults often rest under loose bark or in shaded microhabitats during the day, while their mycetophagous larvae develop in decomposing wood and are highly dependent on the moisture within the wood [11]. Similarly, larvae of Tipuloidea frequently occupy aquatic or semi-aquatic substrates but may also develop in terrestrial habitats, and some are known from decaying damp wood [12]. However, species respond differently, with some showing an association with higher moisture levels, while multiple environmental factors influence others [10].

This variability suggests that ongoing climate change, characterised by rising temperatures and altered precipitation regimes, may significantly impact the abundance and diversity of certain Diptera species. Many nematoceran species are adapted to specific microclimatic conditions and have limited dispersal abilities, making them particularly vulnerable to environmental changes [6,13]. Reduced rainfall and prolonged droughts could accelerate wood drying, limiting the availability of suitable habitats for moisture-dependent species. Given these challenges, understanding the relationship between precipitation and the diversity of wood-associated Diptera is crucial for predicting potential shifts in forest insect communities.

This study aims to evaluate the impact of precipitation, air temperature, and relative humidity on the diversity and abundance of wood-associated nematoceran flies (infraorders Bibionomorpha and Tipulomorpha) in forest reserves across Lithuania. Despite their ecological sensitivity, saproxylic nematocerans remain among the least studied insect groups. The effects of climatic factors on their diversity in Lithuanian forests are mainly unknown, representing a significant research gap.

The present work forms part of a broader project investigating saproxylic insect assemblages in Lithuanian forests. Previous studies within this framework examined species diversity and abundance associated with different tree species [14,15]. However, marked interannual fluctuations in nematoceran abundance prompted questions about the environmental factors driving these variations. By relating abundance dynamics to precipitation and moisture-related variables, this study aims to identify the principal environmental drivers of nematoceran population variability and to complement earlier findings.

We hypothesise that precipitation is the main climatic factor influencing the richness, abundance, and diversity of saproxylic nematoceran flies. By analysing relationships between climatic parameters and community composition, this study seeks to clarify the ecological mechanisms through which precipitation and moisture availability shape nematoceran distribution. These insights will contribute to understanding the potential consequences of climate change, particularly the increasing frequency of drought events, on saproxylic fly assemblages in forest ecosystems.

## 2. Materials and Methods

**Figure 1:**
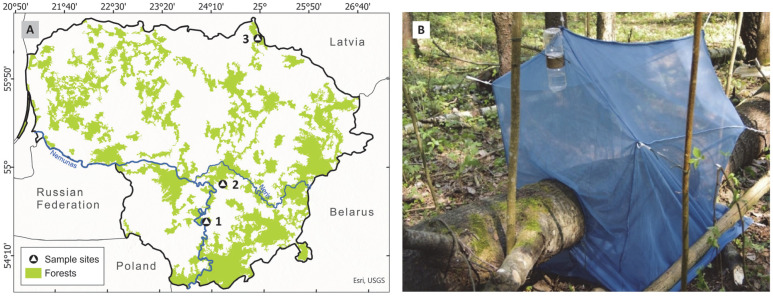
(**A**) Map of Lithuania showing study locations: 1—Punia Šilas strict nature reserve, 2—Būda botanical-zoological reserve, 3—Biržų Giria botanical reserve; (**B**) a trunk-emergence trap placed on a fallen tree trunk of *Populus tremula* within the Būda botanical-zoological reserve, in 2018.

This study was conducted in three nature reserves in Lithuania: Būda Botanical-Zoological Reserve (BR), Biržų Giria Botanical Reserve (BGR), and Punia Šilas strict nature reserve (PR) (Figure 1A, Table 1). The mixed forests of the Būda botanical-zoological and Biržai Giria botanical reserves are dominated by ash (*Fraxinus excelsior* L.), linden (*Tilia cordata* Mill.), and alder (*Alnus glutinosa* (L.) Gaertn.) tree species. The Punia Šilas reserve is one of the oldest forests in Lithuania. It formed a unique ecosystem with old-growth oaks (*Quercus robur* L.), linden (*Tilia cordata* Mill.), maples (*Acer* spp.), pines (*Pinus sylvestris* L.), and other tree species.

To assess the diversity of wood-associated nematocerans, trunk-emergence traps were used following established methodologies [14]. Similar techniques have been widely applied in saproxylic insect research [8,10]. Traps were made from transparent, air-permeable polyester fabric to facilitate insect capture while maintaining ventilation. Each trap covered a 1-m trunk segment, ensuring consistent sampling across all study sites. Tent-like traps were built using ropes and were attached to nearby standing trees, ensuring that there were no fabric folds on the set traps. The front edges of the traps were secured tightly around the fallen tree trunk with ropes, ensuring a complete seal to prevent insects from escaping. The front wall of the trap was elevated by 20 cm above the others, promoting upward insect flight behaviour towards the higher end, where a plastic container containing 99% propylene glycol was suspended to collect trapped insects (Figure 1B).

Five tree species were selected for this study (Table 1). Between 2016 and 2020, emergence traps were set on 31 fallen trees. Due to the inability to determine the exact time of tree death, the wood decay stage was assessed using the description by Stokland [16]. The trunks still had attached bark, and the rot had penetrated up to 3 cm into the wood; therefore, all the logs were classified as stage 2 decay. At this stage of decay, it is still possible to identify the tree species. All traps were set in forests characterised by dense canopy cover and minimal sun exposure. The volume of surrounding dead wood was not quantified, as comparable measurements across different reserve sites were not possible; however, all sites contained dead wood. Sampling occurred from May to November, with traps being emptied every two weeks.

All collected insects were preserved in ethanol and transported to the laboratory for further analysis. Collected specimens were sorted, and nematocerans were used for further identification. Most specimens were identified by the first author using standard identification keys (Keroplatidae, Ditomyiidae, Mycetophilidae [17]; Sciaridae [18]; Limoniidae [19], Tipulidae [20]) and various taxonomic articles and revisions corresponding to individual families. Small species of Sciaridae and Mycetophilidae were slide-mounted and analysed using a microscope. Subsequent analyses focused on the Tipulomorpha and Bibionomorpha (excluding Cecidomyiidae) specimens. Due to the challenges in identifying female specimens, only male Sciaridae and Mycetophilidae were included in the analysis. The collected material is stored in the Zoological Museum of Vilnius University, Lithuania.

Temperature, relative humidity, and precipitation were obtained from the Hydrometeorological Service from the nearest automatic meteorological stations (AMS) to the study locations (Alytus AMS (4452), Kaunas AMS (4351), and Biržai AMS (6451)). For each study period (May to November), the average temperature and relative humidity were calculated based on hourly measurements by summing the daily values and dividing by 24. Precipitation totals were determined by summing hourly precipitation measurements over each month.

To quantify the diversity of saproxylic nematoceran flies in dead wood trunks, we measured species richness (S) and calculated diversity indices: Shannon’s index (H′) (1), which varies from 0 for communities with only a single taxon to high values for communities with many taxa, each with few individuals [21],

(1) $$H^{\prime }=-\sum \frac{n_{i}}{n}ln\frac{n_{i}}{n}$$

and Dominance index (D) (2), which ranges from 0 (all taxa are equally present) to 1 (one taxon dominates the community completely),

(2) $$D=\sum {\left(\frac{n_{i}}{n}\right)}^{2}$$

where n—total number of individuals, n_i_—number of individuals of taxon i, using PAST 4.17 software [22]. Results are shown as mean ± SD. Differences in nematoceran species richness, abundance, and diversity, as well as variations in climatic variables among study years, were assessed using non-parametric Kruskal–Wallis H tests [23]. When significant effects were detected, post hoc pairwise comparisons were performed with Dunn’s tests, applying a Bonferroni adjustment, using PAST 4.17 software; this test reports a z statistic for each comparison [22].

The associations between species richness (S), abundance, and diversity indices of saproxylic nematoceran flies as explained variables and climatic parameters as explaining variables were assessed using four separate stepwise linear regression models that were conducted using SPSS 19. Stepwise selection was employed to identify the most parsimonious model for each biodiversity metric, using the probability of F for entry (*p* < 0.05) and removal (*p* > 0.20). All predictor variables were assessed for multicollinearity using variance inflation factors, with values below 5.0 considered acceptable. Species richness (S) and abundance was log-transformed to improve normality.

## 3. Results

### 3.1. Species Richness and Abundance Across Years

In total, 793 specimens belonging to 10 families were collected during the study period in the years 2016–2020 (Table 2). The most abundant were *Sylvicola cinctus* (Anisopodidae) with 179 specimens, *Scatopsciara calamophila* (Sciaridae) with 73 specimens, and *Gnophomyia viridipennis* (Limoniidae) with 72 specimens. A list of nematoceran species associated with different tree species can be found in the publication by Gorban and Podėnienė [15].

On average, from one trap, 25.58 (±42.38) specimens and 6.55 (±6.44) species were recorded. The highest average abundance was recorded in 2016 with 91 (±71.5) specimens and 14.2 (±4.92) species per trap, and the lowest average abundance was recorded in 2018 with 3.1 (±2.81) and 2.5 (±1.78) species per trap (Table 2).

Kruskal–Wallis tests indicated significant differences in species richness (H = 14.21, *p* = 0.002) and abundance (H = 15.01, *p* = 0.0017), but no significant differences were observed in Shannon’s diversity index (H′) (H = 6.835, *p* = 0.0759) and Dominance index (H = 2.05, *p* = 0.555) between different collecting years. Post hoc tests revealed significant differences in species richness (z = 3.73, *p* = 0.001) and abundance (z = 3.8, *p* < 0.001) between the years 2016 and 2018. Temperature (H = 0.578, *p* = 0.9), precipitation (H = 4.318, *p* = 0.229), relative humidity (H = 4.253, *p* = 0.235) did not significantly differ between the years.

### 3.2. Effects of Weather Variables on Richness and Diversity

**Figure 2:**
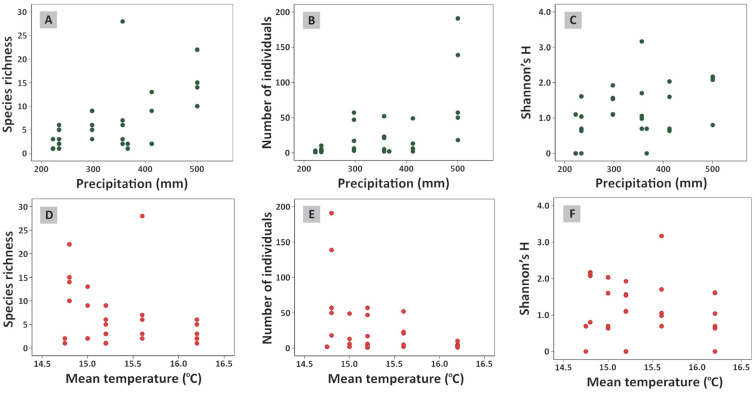
Associations between diversity measures (species richness, number of individuals, and Shannon diversity index (H′)) of nematoceran flies and precipitation (**A**–**C**) and mean air temperature (**D**–**F**).

Species richness (log-transformed) of saproxylic nematoceran flies was significantly associated with precipitation (β = 0.003, β weight = 0.597, SE = 0.001, t = 4.01, *p* < 0.001) (Figure 2A). Mean air temperature (β = 0.306, *p* = 0.133) (Figure 2D) and relative humidity (β = −0.101, *p* = 0.837) were excluded from the model. The model explained 36% of the variance (R^2^ = 0.36, F_1,29_ = 16.05, *p* < 0.001). Similarly, total abundance (log-transformed number of individuals) was significantly positively predicted by precipitation (β = 0.005, β weight = 0.656, SE = 0.001, t = 4.68, *p* < 0.001) (Figure 2B). Mean air temperature (β = 0.206, *p* = 0.287) (Figure 2E) and relative humidity (β = 0.117, *p* = 0.799) were excluded from the model (R^2^ = 0.43, F_1,29_ = 21.91, *p* < 0.001). Shannon’s diversity index (H′) also showed significant positive association with precipitation (β = 0.005, SE = 0.001, t = 2.837, *p* = 0.008) (Figure 2C), while mean air temperature (β = 0.359, *p* = 0.109) (Figure 2F) and relative humidity (β = −0.370, *p* = 0.49) were excluded from the model (R^2^ = 0.22, F_1,29_ = 8.05, *p* = 0.008). Dominance index showed significant negative association with precipitation (β = −0.001, SE = 0.001, t = −2.11, *p* = 0.043), while mean air temperature (β = −0.397, *p* = 0.092) (Figure 2F) and relative humidity (β = 0.405, *p* = 0.472) were excluded from the model (R^2^ = 0.13, F_1,29_ = 4.46, *p* = 0.043).

## 4. Discussion

Although the effects of precipitation, relative humidity, and temperature on different Diptera taxa have been studied [24,25,26,27,28], knowledge of saproxylic nematoceran flies ecology, particularly those within infraorders Bibionomorpha and Tipulomorpha, remains poorly understood. This research investigates the influence of climate-related environmental variables on saproxylic nematoceran communities over a five-year period (2016–2020) in Lithuanian forest reserves. Our results demonstrated that precipitation was the only climatic variable significantly associated with species richness, abundance, and diversity, while air temperature and relative humidity showed no significant effects.

Saproxylic nematocerans are a highly diverse group in forest ecosystems and are essential for decomposition and nutrient cycling [29]. Families, such as Mycetophilidae and Sciaridae, which dominated in our study, are typical indicators of moist, shaded forest habitats rich in fungal resources [11,14,15]. Many species within these families develop as mycetophages in fungal fruiting bodies or within wood colonised by mycelia, while others act as detritivores within decaying bark. Consequently, their population dynamics are expected to reflect the precipitation-driven hydrological balance of forest microhabitats.

Our data showed a decline in both species richness and abundance in 2018, a year characterised by exceptionally low precipitation (266–278 mm less than in 2016) and educed overall moisture levels. Such conditions likely resulted in desiccation of decaying wood, inhibiting larval development and reducing fungal growth—a key food source for mycetophagous larvae. These findings correspond closely with previous research demonstrating that wood-associated Diptera rely heavily on moist microhabitats [4,10,29,30]. Økland et al. [31] reported higher Mycetophilidae richness in wetter Scandinavian forests, while prolonged drought led to sharp declines in species diversity. Similarly, Soszyńska [32] observed that winter activity of Trichoceridae was restricted to near-saturated humidity (~100%), indicating strong physiological dependence on ambient moisture. Moisture not only sustains larval survival but also shapes the microbial and fungal communities within decaying wood, as reduced water content limits fungal growth and indirectly reduces the food available for larvae of saproxylic nematoceran flies [29,33]. The observed increase in nematoceran abundance during wetter years highlights the role of precipitation in maintaining favourable microclimatic conditions and supporting food availability. While other factors, such as local microhabitat variation, tree species composition, or multiyear life cycles, may contribute to abundance differences between years, precipitation appears to be the primary driver of the dynamics. These findings are consistent with prior work showing that saproxylic fly abundance often increases following wetter summers [10].

In contrast to moisture, air temperature did not show a significant influence on nematoceran species richness or abundance, despite a mean increase of approximately 2 °C between 2016 and 2019. Limited research on temperature effects on saproxylic nematoceran flies makes comparisons difficult. Laboratory experiments support this interpretation, as Li et al. [34] showed that *Bradysia odoriphaga* (Sciaridae) can develop in temperatures ranging from 15 to 30 °C, suggesting that some nematocerans can successfully grow within a wide range of temperatures. Field observations elsewhere also indicate complex thermal responses. In a study in North Caroline old-growth forest, species showed decline in extreme warming as temperature rose 2.5 °C above average, as total arthropod abundance decreased by 20.5% per degree of warming, with the most abundant families (over one-third of which were Mycetophilidae) declining by 64.4% [35]. These results suggest that moderate warming may have limited short-term effects on saproxylic nematocerans, but extreme or prolonged heat—especially when coupled with low precipitation—could lead to substantial declines.

Although studies on the impact of temperature on saproxylic nematocerans are lacking, comparative studies on saproxylic beetles further highlight this interaction. Lachat et al. [36] showed that temperature, together with wood availability, strongly influences saproxylic beetle communities, especially in forests with lower amounts of dead wood. Similarly, Goßmann et al. [37] demonstrated that beetle abundance increased with regional temperature and was higher in sun-exposed wood logs compared to shaded ones across multiple Swedish forest regions. However, the lack of a temperature effect in our study suggests that nematoceran flies may be less responsive to thermal variation than to moisture-related factors, reflecting their physiological dependence on humid microhabitats.

The strong sensitivity of saproxylic nematocerans to precipitation has important implications under future climate scenarios. Regional projections for the Baltic region indicate increased temperature variability, more frequent summer droughts, and uneven precipitation patterns [38]. These changes are likely to increase wood desiccation, reduce fungal productivity, and alter the temporal stability of saproxylic habitats. Species with low dispersal ability or narrow water content requirements may experience local extinctions or range contractions [29,39]. Because nematoceran flies contribute to wood decomposition, fungal dispersal, and nutrient cycling, their decline could have cascading effects on forest ecosystem processes. Long-term monitoring that integrates both climatic and microhabitat data is therefore critical for detecting early warning signs of community shifts.

Several limitations of our study should be acknowledged. Some dead wood characteristics, such as trunk diameter, moss coverage, and wood moisture content, were not consistently recorded across study years, making it difficult to compare how microhabitat variables may have influenced species composition. Despite these limitations, the present study substantially advances knowledge of saproxylic nematoceran fly ecology in the Baltic region. By demonstrating that precipitation is the primary determinant of community structure and dynamics, it provides essential baseline data for predicting how climate change may reshape saproxylic fly assemblages. Ultimately, saproxylic nematocerans should be incorporated into long-term biodiversity monitoring programmes as sensitive indicators of forest moisture balance and microclimatic stability.

## Figures and Tables

**Table 1:** Number of emergence traps (n) set on different fallen tree species trunks from which nematocerans emerged across localities during 2016–2020 (only traps with nematoceran emergence included; BR—Būda Botanical–Zoological Reserve, BGR—Biržai Giria Botanical Reserve, PR—Punia Šilas Strict Nature Reserve).

Year	n	Locality	Tree Species (Number of Traps)
2016	5	BR	*Fraxinus excelsior* (4), *Populus tremula* (1)
2018	10	BR	*F. excelsior* (3), *P. tremula* (3)
		BGR	*Alnus glutinosa* (2), *F. excelsior* (2)
2019	7	BR	*F. excelsior* (2), *P. tremula* (3)
		BGR	*A. glutinosa* (1), *F. excelsior* (1)
2020	9	BR	*Tilia cordata* (1), *Quercus robur* (3)
		PR	*T. cordata* (2), *Q. robur* (3)

**Table 2:** Abundance (Individuals_N) and species richness (Taxa_S) of saproxylic nematoceran flies recorded in different years (2016, 2018, 2019, 2020) across Lithuanian forest reserves. Also shown are the average number of species, specimens, Shannon’s diversity index (Shannon_H′), and dominance index (Dominance_D) per trap (±SD), calculated for each year.

Year	2016	2018	2019	2020
Taxa_S	45	19	24	50
Individuals_N	455	31	134	173
Species per trap (±SD)	14.2 (±4.9)	2.5 (±1.8)	5 (±3.2)	8 (±8.4)
Number of individuals per trap (±SD)	91 (±71.5)	3.1 (±2.8)	19.1 (±23.2)	19.2 (±19.3)
Average Shannon’s H′ per trap (±SD)	1.98 (±0.8)	1.31 (±1.6)	0.65 (±0.8)	0.71 (±0.9)
Average Dominance_D per trap (±SD)	0.26 (±0.22)	0.11 (±0.14)	0.28 (±0.36)	0.21 (±0.20)

## Data Availability

The original contributions presented in this study are included in the article. Further inquiries can be directed to the corresponding author.
